# Learning Math: Two Principles to Avoid Headaches

**DOI:** 10.3389/fpsyg.2019.02042

**Published:** 2019-09-06

**Authors:** Felipe Munoz-Rubke, Daniela Vera-Bachmann, Alejandro Alvarez-Espinoza

**Affiliations:** Instituto de Psicología, Universidad Austral de Chile, Puerto Montt, Chile

**Keywords:** math learning, math education, intuitive mathematical knowledge, spatial skills, diagrams, spatial representations

## Introduction

For the last 10 years South American nations have finished in mid to bottom positions in the Programme for International Student Assessment (PISA) math test, significantly behind dozens of countries around the globe. Regrettably, the lack of improvement over the past decade does not depict an optimistic future for this region (OECD, 2017). To reverse this trend, we believe that the recognition and adoption of two key principles could lead to substantial improvements in early math education: first, valuing each student's intuitive math knowledge; and second, focusing on the role that spatial skills play in learning math. We also suggest that both principles could be simultaneously put into practice by utilizing diagrams for teaching early mathematics.

Research shows that multiple interrelated factors explain the poor performance of South American students in mathematics (Cerda et al., 2017). Poverty remains one of the most notable obstacles (Hanushek and Luque, 2003; Kainz, 2019), though other variables at the school level are also relevant. Among these are each school's social climate and educational perspectives (Macneil et al., 2009; Gálvez-Nieto et al., 2015) and each country's public policies in education (Vegas and Petrow, 2007), just to mention a few. Despite this, research shows that the effectiveness of each school is mostly determined by their teachers; teachers' training, knowledge, and beliefs about how to teach mathematics seem to be more relevant than any other factor (Ball et al., 2008; Mapolelo and Akinsola, 2015).

At this level, two principles could be incorporated into early math teaching. Both are supported by considerable evidence and could reduce the sometimes painful experience of learning math. The first principle states that a strong understanding of early mathematics can be built using children's intuitive mathematical ideas as a foundation. This principle mirrors Vygotsky's ideas concerning the bridge that should exist between formal and spontaneous concepts, as the former operates as a zone of proximal development (ZDP) for the latter (Vygotski, 2001). The ZDP corresponds to the distance between current performance under no guidance and potential performance with guidance, and it highlights the linkage between what is currently known and what could be known provided enough support. The second principle indicates that students' spatial skills can influence how much they will get to enjoy and succeed in mathematics. Although there is evidence highlighting the importance of spatial skills in math performance, South American schools have yet to include spatial training in their academic curricula.

Improving math education is important because it could promote the development of South American countries by strengthening their human capital. It is imperative to have more and better professionals in Science, Technology, Engineering, and Mathematics (STEM), who can tackle the challenges that countries face in an increasingly complex and fast-changing economy (Schwab, 2017).

## Role of Children's Intuitive Ideas on Early Math Learning

Everyday mathematics refers to the use of intuitive mathematical notions in real-life contexts. In these situations, people are not directly concerned by specific mathematical principles, but instead use raw intuition to solve applied problems.

There are opposing perspectives on the role that everyday mathematics plays in formal learning. While some researchers see it as a foundation on which students can build meaningful understandings of concepts, others regard it as a source of interference (Carraher and Schliemann, 2002). These opposing views reflect differences in the social valuation of everyday experiences and academic practices (Civil, 2016).

Since the 1980s, researchers have highlighted the role that intuitive mathematical knowledge can play in improving school mathematics, especially in generating more meaningful learning experiences for students (Carraher et al., 1985; Wager, 2012). A seminal study by Carraher et al. (1985) illustrated the use of everyday mathematical knowledge by Brazilian children and adolescents working as street vendors. These participants demonstrated advanced proficiency in solving arithmetic problems when dealing with complex economic transactions, despite their lack of formal mathematical training. Interestingly, these participants made significant mistakes when attempting to solve similar mathematical problems through the traditional algorithmic procedures taught in schools. This disparity in performance made the investigators wonder how it was possible for participants to show high proficiency in one context, and a lack of it in another. In a follow-up study, the investigators showed that meaningful contexts, like those experienced by the street vendors, tend to evoke alternative problem solving strategies based on simple yet powerful heuristics (Carraher et al., 1987).

Previous research has also shown the benefits of Cognitively Guided Instruction (CGI), a professional development program for teachers that underscores the role of children's intuitive ideas in early math education (Carpenter and Fennema, 1992; Carpenter and Franke, 2004). This program does not encourage the application of specific instructional methodologies, but instead stimulates appreciation for the diverse problem solving strategies and distinct understandings that students have of mathematical principles. Upon acknowledging that students are active creators of their own knowledge (Cobb, 1988), CGI teachers ask children to explain their problem solving strategies, familiarize themselves with each children's preferred problem solving approaches, and promote the use of various problem solving methods (Carpenter et al., 1989; Peterson et al., 1989a,b). These behaviors positively correlate with students' problem solving performance.

## Role of Visuospatial Thinking on Early Math Learning

Spatial skills play an important role in STEM disciplines. Longitudinal studies have shown that people with higher spatial skills tend to enjoy, choose, and succeed in STEM areas (Shea et al., 2001; Wai et al., 2009; Lubinski, 2010).

For a long time, spatial abilities were seen as a stable and unmodifiable human trait (Newcombe, 2014). However, multiple investigations contradict this assumption. A recent meta-analysis summarizing the results of more than 200 studies showed that spatial abilities are malleable, that spatial training can promote long-lasting effects, and that training one specific ability can result in the enhancement of other untrained spatial skills (Uttal et al., 2013).

Some studies have focused on the positive direct effects that spatial training can have on mathematical learning. For instance, Cheng and Mix (2012) showed that mental rotation practice can lead to an increase in numerical calculation among 6 and 8 year-olds. In a more natural setting, Lowrie et al. (2017) implemented a 10 week spatial training program in the classrooms of 10-to-12 year-old students. The interventions were implemented by teachers and encompassed the direct training of different spatial skills like mental rotation, spatial orientation, and spatial visualization. Students who underwent this spatial intervention program increased both their spatial and mathematical skills more than the students who were part of the control group. A study by Hawes et al. (2017) used a somewhat different strategy. Instead of training spatial abilities directly, they created spatial games and dynamics to teach mathematical concepts. Their results suggested significant increases in spatial language, spatial reasoning, and numerical comparison following the intervention.

Although we do not yet have a complete understanding of the mechanisms linking spatial abilities and mathematical performance, some studies have already provided hints. A study by Hegarty and Kozhevnikov (1999) suggested that not all types visuospatial representations promote math problem solving. In their study, two visuospatial strategies were contrasted: one based on spatial-schematic imagery and another based on visual-pictorial imagery. Spatial-schematic imagery was defined as the creation of representations that included information about the parts of objects, their spatial relation to other objects, and their respective locations in space. Visual-pictorial imagery was defined as the creation of representations centered on the visual appearance of objects, including properties such as color and shape. Results showed that the use of spatial-schematic strategies, but not of visual-pictorial strategies, was associated with a higher rate of success in mathematical problem solving.

## The Bridge Between Both Principles: Using Diagrams for Math Problem Solving

The two aforementioned principles come together into a single pedagogical practice when diagrams are used to support math problem solving. This is by no means a new idea, as this methodology has been implemented in the educational systems of both Singapore (Ng and Lee, 2009; Kaur, 2018) and Japan (Murata, 2008), countries with outstanding international performances in mathematics.

**Figure 1:**
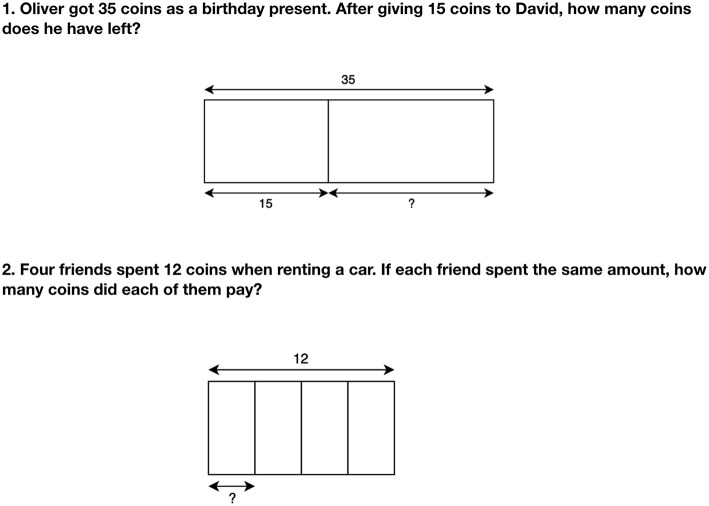
Diagrams showing part-whole models. Both figures are based on Kaur's ideas (Kaur, 2018).

Diagrams are visuospatial representations that depict significant information in a spatial display. Because diagrams are more abstract than objects/manipulatives but more concrete than mathematical symbols, they can provide a valuable bridge between initial and advanced learning stages. In their role as intermediate-level representations, they highlight relationships that could be difficult to spot in higher-level symbolic equations, particularly for novices (for an example, see Figure 1). This is of importance for early math students that are just becoming familiar with the discipline and who often struggle with abstract conceptualizations.

Previous research shows that diagrams encourage the use of alternative, intuitive problem solving strategies. For instance, they can facilitate the application of children's intuitive mathematical ideas during early arithmetic lessons and more advanced algebra lessons (Edens and Potter, 2008; Murata, 2008; Chu et al., 2017). For instance, in a study that included a brief intervention targeted at teaching seventh-grade American students to use diagrams to solve algebra problems, Chu et al. (2017) found that diagrams favored the utilization of informal problem solving strategies and led to significant gains in solving accuracy.

The role of diagrams as visual-spatial representations that favor the use of intuitive problem solving strategies is stressed by concreteness fading, a theory of instruction based on the ideas of Bruner (1966) and subsequently developed by Fyfe and Nathan (2018). This theory suggests that the best way to achieve a deeper understanding of a concept is to first ground it at a concrete level, and to then progressively expose the learner to more abstract instances of it. In the first representational level, interactions with objects and places represent the relevant concept (e.g., learning subtraction by counting apples). During the second representational level, students deal with representations that are more abstract but that still resemble concrete objects, places, and their relationships (e.g., learning subtraction by using diagrams). The third representational level corresponds to the symbolic stage, in which the representations have no obvious relation with objects and spaces (e.g., learning subtraction by using numbers).

Similar ideas applied to learning geometry have been endorsed by Battista (2007), who suggests that students should move from visualization, to abstraction, to formal deduction, until reaching higher mathematical rigor.

## Conclusion

South American early math education could be improved through the adoption of these two central principles. The first principle indicates that learning formal concepts becomes more meaningful when teachers integrate what children already know. The second principle indicates that spatial abilities have a strong and positive effect on both the motivation to learn math and math performance itself. The evidence points out that spatial training at an early age can lead to improvements in the mathematical performance of students. While most early education programs consider the development of language and math skills, the development of spatial thinking has not received systematic attention.

Both principles are integrated into math problem solving through the use of diagrams. Diagrams, as intermediate representations between the concrete and the abstract, are highly effective in the development of mathematical learning. The attractiveness and simplicity of diagrams can make it easier for children to build meaning around mathematical activity. That is, students can link abstract concepts with elements of their own experience in a way that allows the appropriation of concepts.

Although the success of Singapore and Japan in mathematics is certainly the result of multiple features, evidence suggests that incorporating the methodical use of diagrams during math lessons could have played a role. These initiatives were possible due to the existence of public policies in education that encouraged new practices guided by scientific evidence. South American countries, in contrast, have a notable gap between public policies, scientific evidence, and educational practices. This is important because public education has a strong impact on a country's social and economic development, and there is no doubt that well-formed human capital tends to generate innovation, a crucial factor for competing in a globalized world.

## Author Contributions

All team members contributed to this project. FM-R, DV-B and AA-E wrote and reviewed the final manuscript.

### Conflict of Interest Statement

The authors declare that the research was conducted in the absence of any commercial or financial relationships that could be construed as a potential conflict of interest.
